# Long-Term Administration of Abacavir and Etravirine Impairs Semen Quality and Alters Redox System and Bone Metabolism in Growing Male Wistar Rats

**DOI:** 10.1155/2021/5596090

**Published:** 2021-07-29

**Authors:** Agnieszka Matuszewska, Beata Nowak, Wojciech Niżański, Maria Eberhardt, Kinga Domrazek, Anna Nikodem, Benita Wiatrak, Krzysztof Zduniak, Kamil Olejnik, Anna Merwid-Ląd, Tomasz Tomkalski, Diana Jędrzejuk, Ewa Szeląg, Marzenna Podhorska-Okołów, Aleksandra Piotrowska, Izabela Jęśkowiak, Agata Heinrich, Maria Rutkowska, Wojciech Dziewiszek, Tomasz Sozański, Joanna Kwiatkowska, Paulina Jawień, Marek Bolanowski, Adam Szeląg

**Affiliations:** ^1^Department of Pharmacology, Wrocław Medical University, Wrocław 50-367, Poland; ^2^Department of Reproduction and Clinic of Farm Animals, Wrocław University of Environmental and Life Sciences, Wrocław 50-375, Poland; ^3^Department of Small Animal Diseases and Clinic, Lab of Small Animal Reproduction, Institute of Veterinary Medicine, Warsaw University of Life Sciences, Warsaw 02-787, Poland; ^4^Department of Mechanics, Materials and Biomedical Engineering, Wrocław University of Science and Technology, Wrocław 50-370, Poland; ^5^Department of Pathology, Wrocław Medical University, Wrocław 50-367, Poland; ^6^Department of Endocrinology, Diabetology and Internal Medicine, Tadeusz Marciniak Lower Silesia Specialist Hospital-Centre for Medical Emergency, Wrocław 54-049, Poland; ^7^Department of Endocrinology, Diabetes and Isotope Therapy, Wrocław Medical University, Wrocław 50-367, Poland; ^8^Department of Maxillofacial Orthopaedics and Orthodontics, Wrocław Medical University, Wrocław 50-425, Poland; ^9^Department of Ultrastructure Research, Wrocław Medical University, Wrocław 50-367, Poland; ^10^Department of Histology and Embryology, Department of Human Morphology and Embryology, Wrocław Medical University, Wrocław 50-367, Poland

## Abstract

Highly active antiretroviral therapy (HAART) is used in HIV-infected patients. Alongside the prolongation of patients' life, adverse side effects associated with long-term therapy are becoming an increasing problem. Therefore, optimizing of HAART is extremely important. The study is aimed at evaluating the toxicity of abacavir and etravirine in monotherapy on the reproductive system, liver, kidneys, and bones in young, sexually mature, male rats. Thirty-six 8-week-old male Wistar rats randomized into three 12-animal groups received either normal saline (control), abacavir 60 mg/kg (AB group), or etravirine 40 mg/kg (ET group) once daily for 16 weeks. Semen morphology, oxide–redox state parameters (MDA, SOD, catalase, GPx, glutathione, GSH/GSSG ratio) in tissue homogenates (testes, liver, kidneys), and serum samples were studied. In bones, microcomputed tomography and a four-point bending test were performed. Total sperm count, sperm concentration, motility, and sperm morphology did not differ significantly in AB or ET groups compared to the control. In the flow cytometry of semen, an increased percentage of cells with denatured DNA was noticed for both tested drugs. However, no significant changes of oxide–redox state in testicular homogenates were found, except of increased SOD activity in the AB-receiving group. Additionally, ET significantly altered catalase and GPx in the liver and SOD activity in kidneys. Abacavir decreased catalase in the liver and GSH levels in kidneys. AB caused significant changes to bone microarchitecture (bone volume fraction, trabecular number, connectivity density, total porosity) and increased Young's modulus. Etravirine had a greater impact on macrometric parameters of bones (tibial index, mid-tibial diameter, femur length). After 4 weeks in the ET group, a lower 1,25-dihydroxyvitamin D_3_ serum concentration was found. The results showed that abacavir and etravirine disturb oxidative stress. An increase in the percentage of sperms with chromatin damage suggests decreased fertility in rats receiving the studied drugs. Both drugs affected bone formation in growing rats. Additionally, etravirine disturbed vitamin D metabolism.

## 1. Introduction

According to the World Health Organization (WHO) statistics, there are about 37.9 million people infected with the human immunodeficiency virus (HIV). Despite increased public awareness, many awareness-raising campaigns, and better health education, there are about 1.7 million new HIV cases every year [1]. Antiretroviral therapy made it possible to decrease the viral load below the sensitivity limit of diagnostic tests, caused an increase in the level of CD4 lymphocytes, and reduced the incidence of opportunistic infections and the development of full-blown acquired immunodeficiency syndrome (AIDS). As a consequence, mortality in HIV patients has decreased significantly [2–4]. However, drug-induced side effects associated with long-term antiretroviral therapy are becoming a growing concern [2, 3, 5]. HIV therapy involves highly active antiretroviral therapy (HAART), meaning that patients are treated with drug combinations. The identification of the numerous adverse effects of a single drug becomes problematic.

The proposed mechanism of toxic action of HAART on testes, sperm, bones, liver, kidneys, and other tissues is complex and not fully understood. In hepatotoxicity, e.g., hypersensitivity with acute hepatic necrosis, metabolic-host mediated injury, or mitochondrial toxicity are postulated. Kidney injury may depend on the action on organic anion transporters in proximal tubules and the accumulation of active drugs in this structure. In many tissues, the imbalance of oxidative stress parameters (pro- and antioxidants) with the resultant oxidation of biomolecules is an important trigger mechanism leading to tissue damage and dysfunction [6–8].

Widespread access to antiretroviral therapy has led many perinatally HIV-infected children to reach adolescence and adulthood [9, 10]. They become sexually active and make decisions about their reproductive health. It is estimated that about 75% of persons who are infected with HIV are in the reproductive period of life [11]. The reproductive system, especially of males, is overly sensitive to many xenobiotics. Drugs are given in a long-term schedule [6]. Iyer et al. reported that HIV status and HAART administration were associated with subfertility both in males and females [12]. In males, HAART was found to impair semen quality [13]. Kehl et al. detected a lower ejaculate volume as well as less slow progressive and more abnormally shaped spermatozoa in HAART-receiving patients [13]. Other authors reported altered mitochondrial DNA and intensified oxidative stress in HAART-receiving males that negatively impact testicular functions [14]. Concentrations of antiretroviral drugs in the seminal plasma vary between drugs. They are the highest for nucleoside analogues and lowest for protease inhibitors. Abacavir and etravirine reach a high concentration in the seminal plasma [15, 16] as they easily cross the blood-testis barrier [16, 17]. On one hand, a high concentration of an antiretroviral drug in the seminal plasma is favorable, because the drug may suppress viral replication in semen and prevent the sexual transmission of the infection. On the other hand, a higher concentration of the drug in testes increases the risk of gonadal toxicity. Most men and women with HIV desire to have children [18]. Therefore, it is extremely important to investigate the effect of drugs given for a long time to young patients on the reproductive system and fertility.

Osteoporosis is a generalized disease of bones with disorders of bone microarchitecture and low bone mass, leading to increased bone fragility [19]. Osteoporosis is a silent disease with a long-lasting symptomless period, and the first clinical symptom is often an osteoporotic fracture [20]. Osteoporotic fractures may be spontaneous or follow a minor trauma. Spine, hip, distal forearm, or rib fractures are the most characteristic for patients with osteoporosis [21, 22]. The occurrence of a bone fracture indicates the advanced stage of the disease, decreases the quality of life, and is the cause of disability and premature death [20, 23]. Therefore, not only the prophylaxis of osteoporosis and osteoporotic fractures but also the recognition of drugs causing osteoporosis seems to be very important.

In HIV patients, the risk of osteoporosis is 3.7 times higher than in the general population [24]. Many studies also suggest a significantly increased risk of fracture in people with HIV [25, 26], reaching 12.8/1,000 people/year [25]. One of the important factors influencing bone metabolism and properties in HIV-positive patients may include the administration of antiretroviral drugs. Retrospective and prospective studies have shown 2%-6% decreases in bone mineral density (BMD) in the first 2 years after the combined antiretroviral therapy started [27]. In children, a reduction in bone mass gain and a decrease in peak bone mass have been observed, resulting in an increased incidence of osteoporosis and bone fractures later in their lives [28, 29].

Abacavir can be used in children from 4 months of age [30] and etravirine in children from 2 years of age [31]. In vitro studies suggested that abacavir and etravirine may affect bone cell differentiation [32]. As antiretroviral drugs are given to humans as part of HAART, it is impossible to assess the testicular toxicity and the influence of a single drug on bone metabolism in humans. Single antiretroviral agents are used in various HAART schedules and are continuously updated [33–35]. It means that the HAART schedule is not fixed, but changes over time [33–35]. In order to choose the optimal HAART drug combination, the efficacy of drugs and current knowledge about viral mutation and drug resistance is just as important as the adverse effects [36, 37]. The schedule is also personalized due to comorbidities and the risk of drug-drug interactions so the potential for a single agent to cause selective tissue toxicity (not only for drug combinations) may be very important in terms of making clinical decisions [38, 39].

Rats are very often used as the animal experimental model for the assessment of the impact of drugs on the male reproductive system [40–42], bones [43, 44], and as well as for the evaluation of direct tissue toxicity [45, 46]. Laboratory methods and reagents are available for measurements of hormone levels and immunological studies in rats, ensuring repeatability and facilitating the comparison of results. Rat models allow scientists to eliminate some variations observed in human trials (e.g., possibility for monotherapy, homogenous groups, lack of concomitant diseases, identical environmental conditions, and diet). The study was conducted in 8-week-old, young adult, male rats. Male rats are sexually mature at the age of six weeks because they develop rapidly during infancy [47, 48]. The most active growth of male rats is during the first 8 months of life [49]. Therefore, our study was conducted between the 8^th^ and 24^th^ week of their lives.

The aim of this study was to evaluate the influence of long-term abacavir or etravirine monotherapy on testicular toxicity and bones. As hepato- and nephrotoxicity often limit drug usage, the authors also decided to investigate the effect of these drugs on the liver and kidneys.

## 2. Materials and Methods

### 2.1. Ethical Statement and Animals

The study was approved by the Local Ethics Committee for Animal Experiments in Wroclaw at the Ludwik Hirszfeld Institute of Immunology and Experimental Therapy of the Polish Academy of Sciences (Approval No. 38/2019). All animal procedures during the study followed the ethical standards and practices of the institution where the study was conducted [50].

Rats were bred and housed at the Animal Laboratory of the Wroclaw Medical University. During the study, animals were housed two per cage with enrichment products (shelters, aspen gnawing sticks, cardboard rolls) with a 24-hour cycle (controlled 12 hours in the light/12 hours in the dark), at an ambient temperature of 22°C, with ventilation, with free access to water, and standard certified animal feed (Altromin 1324, Germany). The feed contained 0.7% calcium, 0.5% phosphorus, and 600 U.I. vitamin D3/kg.

### 2.2. Study Design

Thirty-six male 8-week-old Wistar rats were randomly divided into three groups (twelve rats each). The groups were organized as follows:

- The control group (group C) received a 0.9% saline solution in a volume of 4 ml/kg
- The AB group received abacavir at a dose of 60 mg/kg (Ziagen, Santa Cruz Biotechnology, USA) suspended in a 0.9% saline solution in a volume of 4 ml/kg
- The ET group received etravirine at a dose of 40 mg/kg (Etravirine, Biosynth Carbosynth, UK) suspended in a 0.9% saline solution in a volume of 4 ml/kg

According to the recommended 3Rs rule [50], to minimize the number of animals in the study, the impact of drugs used in monotherapy was evaluated, not the drug combination. The single dose of abacavir and etravirine for rats was calculated according to the recommendations for converting doses from humans to rats [51] (Table 1). Tested antiretroviral drugs and/or normal saline were administered once daily for 16 weeks intragastrically via a gastric tube.

**Figure 1:**
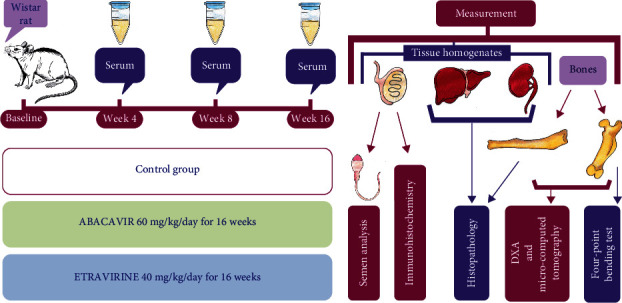
General design of the study.

The study design is presented in Figure 1.

After 4 and 8 weeks of the study, blood samples from the tail vein were taken. Eight hours before their blood was taken, the animals were deprived of the feed. Markers of bone turnover must be assessed after a period of fasting. Additionally, to minimize the impact of calcium and vitamin D-containing food on the evaluation these blood parameters, food deprivation was necessary.

After 16 weeks of the study, the rats were anesthetized by intraperitoneal injections of ketamine (60 mg/kg) and xylazine (10 mg/kg), and blood samples were taken via a cardiac puncture. After that, the rats were euthanized by cervical vertebrae dislocation in deep anesthesia. The epididymis, testes, liver, kidneys, and bones (tibia and femur) were dissected immediately and carefully cleaned of surrounding tissues.

Spermatozoa were collected by epididymal slicing with a scalpel blade according to the method used by Martinez-Pastor et al. [52]. Epididymis was dissected from testicles, cleaned of surrounding tissues, and placed on glass Petri dishes containing 1 ml of the HTF medium (Human Tubal Fluid). Multiple incisions of the epididymal cauda were performed to extract sperm cells from tubules. After slicing, Petri dishes were placed on the heating stage for 10-minute incubation. Subsequently, epididymal tissues were removed, and white fluid containing spermatozoa was analyzed.

The testes, liver, kidneys, right tibia, and right femur were weighed and measured. Then, the right testis, right liver lobe, and right kidney were stored at -80°C and later homogenized as described below. The right tibia and right femur were evaluated densitometrically and with microcomputed tomography (mCT). Then, the biomechanical properties of the right femur were measured using a four-point bending test. The left testis, left liver lobe, left kidney, and left tibia were stored in 10% neutral buffered formalin for histopathological examination.

### 2.3. Serum Parameters

Blood samples were centrifuged at 4,000 g at 4°C for 10 minutes in the MPW-350R laboratory centrifuge (MPW Med. Instruments, Poland). The obtained serum was frozen at -80°C until the proper measurements were made. For the serum, the following parameters were assessed using purchased ELISA kits in accordance with the manufacturers' instructions: luteinizing hormone (LH), follicle-stimulating hormone (FSH), testosterone, sex hormone-binding globulin (SHBG), inhibin B, estradiol, thyroid-stimulating hormone (TSH), prolactin, aminoterminal propeptide of type I procollagen (PINP), osteoclast-derived tartrate-resistant acid phosphatase form 5b (TRACP), sclerostin, Dickkopf-related protein 1 (DKK1), osteoprotegerin (OPG), 25-hydroxyvitamin D (25-OH-D), 1,25-dihydroxyvitamin D_3_ (1,25-(OH)_2_-D_3_), parathormone, and aspartate aminotransferase (AST) (Bioassay Technology Laboratory China: Rat Luteinizing Hormone ELISA kit EA0013Ra, Rat Follicle-stimulating Hormone ELISA kit REF E0182Ra, Rat Testosterone ELISA kit REF E0259Ra, Rat Sex Hormone-binding Globulin ELISA kit E0646Ra, Rat Inhibin B ELISA kit REF E0728Ra, Rat Estradiol ELISA kit REF E0174Ra, Thyroid-Stimulating Hormone ELISA kit REF E0180Ra, Rat Prolactin ELISA kit E0190Ra; Immunodiagnostic Systems Limited UK: Rat/Mouse PINP EIA REF AC-33F, Rat TRAPTM ELISA REF SB-TR102; Cloud-Clone Corp. USA: ELISA kit for Sclerostin SEC864Ra; ELISA kit for Dickkopf Related Protein 1 SEA741Ra, ELISA kit for Osteoprotegerin SEA108Ra; Bioassay Technology Laboratory China: Rat 25-hydroxyvitamin D ELISA kit E1445Ra, Rat 1,25-dihydroxyvitamin D_3_ ELISA kit E0000Ra; Cloud-Clone Corp. USA: ELISA kit for Parathyroid Hormone CEA866Ra; ELK Biotechnology China: Rat AST ELISA Kit Cat. ELK5635).

Serum total calcium, inorganic phosphorus, and creatinine levels were determined in a certified laboratory using an Alinity C instrument with Abbott (calcium G75615R01 B7P57P; phosphorus G84652R01 B8P40P; creatinine G75642R02 B7P99P).

### 2.4. Macrometric Parameters

The testes, liver, kidneys, right tibia, and right femur were weighed using the RADWAG AS 60/220/C/2 (Poland) electronic weighing scale. Based on the obtained measurements, the testicular index, hepatic index, renal index, tibial index, and femoral index were calculated according to the following formulas:

(1) $$\begin{array}{c} \text{Testicular index}=\frac{\text{mass of testes }\left[\text{g}\right]}{\text{body weight }\left[\text{g}\right]}\times 100\%, \end{array}$$

(2) $$\begin{array}{c} \text{Hepatic index}=\frac{\text{liver mass }\left[\text{g}\right]}{\text{body weight }\left[\text{g}\right]}\times 100\%, \end{array}$$

(3) $$\begin{array}{c} \text{Renal index}=\frac{\text{mass of kidneys }\left[\text{g}\right]}{\text{body weight }\left[\text{g}\right]}\times 100\%, \end{array}$$

(4) $$\begin{array}{c} \text{Tibial index}=\frac{\text{tibia mass }\left[\text{g}\right]}{\text{body weight }\left[\text{g}\right]}\times 100\%, \end{array}$$

(5) $$\begin{array}{c} \text{Femoral index}=\frac{\text{femur mass }\left[\text{g}\right]}{\text{body weight }\left[\text{g}\right]}\times 100\%. \end{array}$$

Measurements of bone length were made using the electronic calliper with the 0.01 mm resolution (Pro Sp. z o.o., Poland).

### 2.5. Semen

#### 2.5.1. Spermatozoa Recovery

The epididymis from eight randomly chosen rats in each group was examined. Spermatozoa were collected by epididymal slicing with a scalpel blade according to the method described by Martinez-Pastor et al. [52]. The epididymis was dissected from testicles, cleaned of surrounding tissues, and placed on glass Petri dishes containing 1 ml of the HTF medium (Human Tubal Fluid). Multiple incisions of the epididymal cauda were performed to extract sperm cells from tubules. After slicing, Petri dishes were placed on the heating stage for 10-minute incubation. Subsequently, epididymal tissues were removed, and white fluid containing spermatozoa was analyzed.

#### 2.5.2. Motility Assessment

Subjective motility was determined immediately after removal of the sliced epididymal tissue. A drop (10 *μ*l) of spermatozoa-rich fluid was placed on a glass slide and covered with a cover slide. The evaluation was performed using the phase-contrast microscope (Nikon Eclipse E200; 200× zoom) with a warm stage by two independent researchers, and the mean value was calculated.

#### 2.5.3. Morphology Evaluation

Morphology was assessed after staining with Giemsa stain according to the modified Watson method [53]. Spermatozoa were classified as normal or possessing one of the abnormalities: proximal droplet (%), distal droplet (%), head abnormalities (%), detached head (%), acrosome abnormalities (%), midpiece defects (%), “Dag-like” defect (%), bent tail (%), and coiled tail (%) [54].

#### 2.5.4. Computer-Assisted Sperm Analysis (CASA)

The computer-assisted analysis of sperm concentration was performed using HTM IVOS ver. 12.2 (Hamilton Thorne Biosciences, Beverly, MA, USA). The spermatozoa-rich fluid in the amount of 10 *μ*l was suspended in 40 *μ*l of the HTF extender with 50 *μ*l of IDENT Stain (Hamilton Thorne) and incubated for 5 minutes at 37°C before evaluation. The analysis was carried out using IDENT settings (frames: 60; No. of frames: 40; minimum contrast: 30; minimum cell size: 4; cell size: 13; cell intensity: 75) [55].

#### 2.5.5. Assessment of the Function and Structure of Sperm Cells by Flow Cytometry

Sperm cell functionality was evaluated using fluorescent staining and flow cytometry analysis. Flow cytometric analyses were performed on the Guava EasyCyte 5 (Merck KGaA, Darmstadt, Germany) cytometer. The fluorescent probes used in the study were excited by the Argon ion 488 nm laser. Acquisitions were made using GuavaSoft™ 3.1.1 (Merck KGaA, Darmstadt, Germany). Nonsperm events were gated out based on scatter properties and not analyzed. A total of 10,000 events were analyzed for each sample. The following features were assessed: membrane integrity, acrosome integrity, mitochondrial activity, lipid peroxidation, apoptosis and membrane lipid disorder, and chromatin status [55, 56].

The membrane integrity of rat spermatozoa was assessed using SYBR-14 stain combined with propidium iodide (PI) (Life Technologies Ltd., Grand Island, NY, USA). The sperm-rich fluid in the amount of 300 *μ*l was incubated in the dark for 10 minutes with 5 *μ*l of the SYBR-14 working solution (0.1 *μ*l of SYBR14+ 4.9 *μ*l of TRIS III extender). The analysis was performed after 3 minutes of incubation with 3 *μ*l of PI. The spermatozoa with intact membranes emitted green fluorescence. The cells with red fluorescence were classified as dead [56].

Acrosome integrity was assessed by lectin PNA stain from the Arachis hypogaea Alexa Fluor® 488 conjugate (Life Technologies Ltd., Grand Island, NY, USA). Diluted samples were mixed with 10 *μ*l of the PNA working solution (1 *μ*g/ml) and incubated for 5 minutes at room temperature in the dark. Before the analysis, the samples were washed, and 3 *μ*l of PI was added [55].

Mitochondrial activity was determined using the JC-1 dye (Life Technologies Ltd., Grand Island, NY, USA). The spermatozoa-rich fluid in the amount of 500 *μ*l was stained with 0.67 *μ*l of the JC-1 stock solution (3 mM stock solution of JC-1 in DMSO). The samples were incubated for 20 minutes at 37°C in the dark. The sperm cells emitting orange fluorescence were classified as having high mitochondrial activity. The spermatozoa emitting green fluorescence were defined as those with low mitochondrial activity [55].

Lipid peroxidation (LPO) was evaluated by dyeing using the C_11_-BODIPY^581/591^ fluorescent lipid probe (Life Technologies Ltd., Grand Island, NY, USA). One *μ*l of 2 mM C_11_-BODIPY^581/591^ in ethanol was added to a diluted sperm-rich fluid and incubated for 30 minutes at 37°C in the dark. Subsequently, centrifugation at 500 × g for 3 minutes was performed, and the sperm pellets were resuspended in 500 *μ*l of the HTF extender. To determine viability, the spermatozoa were stained with PI and incubated for 5 minutes at room temperature. The sperm cells emitting orange fluorescence (nonoxidized state of C_11_-BODIPY^581/591^) were defined as live cells without LPO [55].

Apoptosis and membrane lipid disorder were evaluated with the YO-PRO-1 dye (25 *μ*M solution in DMSO) (Life Technologies Ltd., Grand Island, NY, USA) [48]. One *μ*l of YO-PRO-1 stain (final concentration: 25 nM) was added to 1 ml of a diluted spermatozoa-rich fluid (500 *μ*l of HTF and 500 *μ*l of spermatozoa solution). After 10-minute incubation, 3 *μ*l of PI was added before cytometric analysis. The cells showing green fluorescence were classified as YO-PRO-1 positive. The spermatozoa that remained unstained were categorized as living population [56].

Chromatin status was established using the acridine orange dye (AO, Life Technologies Ltd., Grand Island, NY, USA). The spermatozoa-rich solution (100 *μ*l) was subjected to brief acid denaturation by adding 200 *μ*l of the lysis solution (Triton X-100 0.1% (v/v), NaCl 0.15 M, HCl 0.08 M, pH 1.4). After 30 seconds, 600 *μ*l of the AO solution (6 *μ*g AO/ml in a buffer: citric acid 0.1 M, Na_2_HPO_4_ 0.2 M, EDTA 1 mM, NaCl 0.15 M, pH 6) was added. The analysis was performed after 3 minutes of incubation. The spermatozoa with high DNA stainability emitted green fluorescence (HDS). The sperm cells emitting red fluorescence were considered a population of cells characterized by a high DNA fragmentation index (DFI) [55].

### 2.6. Tissue Homogenates

#### 2.6.1. Reproductive Hormones in Tissue Homogenates

In testicular homogenates, testosterone, estradiol, and inhibin B concentrations were evaluated using ELISA kits: Rat Testosterone ELISA REF E0259Ra, Bioassay Technology Laboratory; Rat Estradiol ELISA Kit REF E0174Ra, Bioassay Technology Laboratory; Rat Inhibin B ELISA kit REF E0728Ra Bioassay Technology Laboratory; China.

#### 2.6.2. Preparation of Tissue Homogenates for Sex Hormone Assessment

To 160 mg of testis tissue, 800 *μ*l of the cooled (-20°C) methanol: propanol (1 : 1) mixture was added. The Pro250 homogenizer (PRO Scientific Inc., Oxford, CT, USA) was used. After homogenization, the samples were centrifuged for 10 minutes at 15,000 × g, and the supernatant was collected.

#### 2.6.3. Redox Status in Tissue Homogenates (Figure 2)

Oxide–redox state parameters such as malondialdehyde (MDA), superoxide dismutase (SOD), catalase, glutathione peroxidase (GPx), and glutathione were assessed in homogenates from the testes, liver, and kidneys. The following kits, Rat Malondialdehyde ELISA Kit E0156Ra, Bioassay Technology Laboratory China; Superoxide Dismutase Assay Kit No 706002, Cayman; Catalase Assay Kit No 707002, Cayman; Glutathione Peroxidase Assay Kit No 703102, Cayman; Glutathione Assay Kit No 703002, Cayman; USA, were used.

#### 2.6.4. Preparation of Tissue Homogenates for Redox State Assessment

The MDA concentration was measured in the homogenates that were prepared in the same way as for measurements of reproductive hormones. The oxide–redox state was assessed from the tissues of the liver, kidney, and testes. For all tested parameters, the tissues were prepared in the buffers recommended by the manufacturer at a concentration of 10%. Superoxide dismutase activity was assessed in a tissue solution in the 20 mM HEPES buffer, pH 7.0, supplemented by 1 mM EDTA, 210 mM mannitol, and 70 mM sucrose. Catalase activity was evaluated in the homogenates prepared in 50 mM potassium phosphate, pH 7.0, containing 1 mM EDTA. Glutathione peroxidase activity in the tissues was measured in the 50 mM Tris-HCl, pH 7.5, 5 mM EDTA, and 1 mM DTT solution. The last parameter—glutathione—was analyzed in the same homogenate solution as the one prepared to evaluate catalase activity. In the latter case, after collecting the supernatant, 0.1 g/ml of MPA was added for 5 minutes at room temperature; then, the samples were centrifuged again at >2,000 g for at least two minutes, and the collected supernatant was stored at -20°C until assayed. The other centrifugation supernatants were stored at -80°C until assayed.

#### 2.6.5. Immunohistochemical Examination (IHC) of Testes

Slides were deparaffined and rehydrated, and antigen retrieval was carried out by boiling the sections in EnVision FLEX Target Retrieval Solution pH 9 using a PTLink-20 minutes, 97°C (Dako, Glostrup, Denmark). The visualization of the studied antigen was performed using the EnVision FLEX+, Mouse, High pH System (Dako) according to the manufacturer's instructions. The detection of the minichromosome maintenance 7 protein (MCM-7) antigen (1 : 50, Leica Novocastra, Wetzlar, Germany) and the glutathione antigen (1 : 100, Abcam, Cambridge, UK) was conducted for 20 minutes at room temperature. Then, slides were incubated with secondary antibodies conjugated with horseradish peroxidase (EnVision FLEX/HRP-20 minutes at room temperature). Finally, the sections were counterstained with EnVision FLEX Hematoxylin (Dako) dehydrated in graded ethanol concentrations (70%, 96%, 99.8%) and in xylene and closed in Dako Mounting Medium (Dako). The primary antibody was diluted in the EnVision FLEX Antibody Diluent background-reducing reagent (Dako).

#### 2.6.6. Expression of Minichromosome Maintenance 7 Protein (MCM-7)

The IHC of MCM-7 sections of testes was evaluated in a blinded way under the BX-41 light microscope (Olympus, Tokyo, Japan). For the evaluation of MCM-7-positive cells, three fields with the highest number of cells showing a positive reaction were selected. The evaluation was performed by counting brown-labeled nuclei at ×400 magnification. The percentage of positive cells in each field was assessed according to the following formula: (positive cells/all cells) × 100%.

#### 2.6.7. Expression of Glutathione in IHC in Testes

For the evaluation of the cytoplasmic expression of glutathione levels, the semiquantitative method—immunoreactive score (IRS) according to Remmele and Stegner [57–59]—was applied, and samples were evaluated in the blinded way. The scale is based on the percentage of cells showing a positive reaction (0 points—no cells with a positive reaction; 1 point—1-10% cells with a positive reaction; 2 points—11-50%; 3 points—51-80%; 4 points—>80% cells) as well as on the intensity of the reaction color (0—no reaction; 1—weak; 2—moderate; 3—strong). The IRS final score is the result of multiplying the score obtained from the percentage of cells with a positive reaction by the score of the reaction intensity and is in the range of 0-12 [57–59].

#### 2.6.8. Liver and Renal Histology

The liver and kidneys were fixed in 10% neutral buffered formalin. The dehydration process was then performed using graduated concentrations of ethanol and ethanol-xylene. The final solution was pure xylene. The tissue was infiltrated with appropriate purified paraffin. From the fixed liver and kidney tissues in paraffin, slices 4 *μ*m thick were cut with a microtome and standard stained with hematoxylin and eosin. The preparations were assessed under the Olympus BX50 light microscope equipped with the Zeiss Axiocam 208 color microscope camera using Labscope.

#### 2.6.9. Dual-Energy X-Ray Absorptiometry (DXA)

The right tibia and right femur were examined densitometrically. The tests were performed on Hologic DXA equipment (Hologic Discovery W 81507, Marlborough, USA) using software for small animals. The scanner was calibrated daily with the phantom supplied by the manufacturer. Bone mineral density (BMD) results were obtained as grams of mineral content per square of bone area (g/cm^2^) [60].

#### 2.6.10. Micro-X-Ray Computed Tomography

Structural properties were measured using the SkyScan 1172, Bruker® computed microtomograph. Each sample was registered with a resolution of 9 *μ*m, a voltage of 74 kV at 133 *μ*A, using a 0.5 mm Al filter. 3D structural properties were measured using CTAn (CTAn, Bruker). For each long bone (tibia and femur), measures were taken for 2 areas: the cancellous bone and cortical bone.

**Figure 3:**
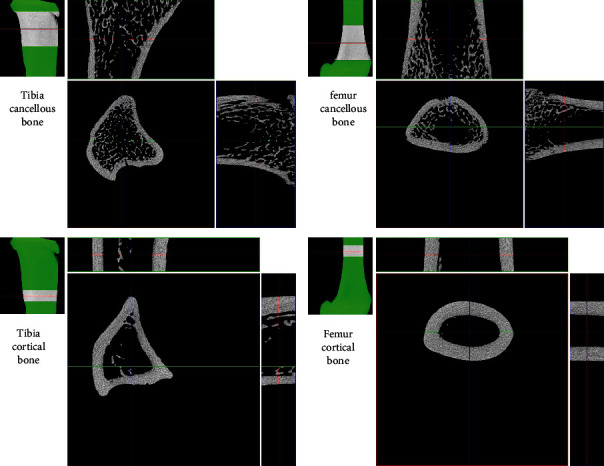
Choice of the region of interest (ROI) for measurements in the cancellous and cortical bone of (a) the proximal metaphysis of the tibia and (b) the distal metaphysis of the femur.

Before 3D morphometric analyses, the images were aligned with the major bone axis, and a set of trabecular and cortical regions of interest (ROIs) was selected. The selection of ROIs (Figure 3) was based on the commonly accepted procedure used for small animals [61–63]. It was carried out in the CTAn programme (Bruker®) based on an automated algorithm prepared in accordance with the MCT-003 method note [64]. The growth plate was used as an anatomical referent to determine trabecular and cortical regions used in the estimation of 3D structural properties. To select the location of the trabecular region, an offset of 100 slices from the growth plate towards the metaphysis, where the trabecular ROI starts, was applied. In other words, an offset is a number of cross-sections between the growth plate and the start of the trabecular ROI. From this location, an extent of 400 slices defines the trabecular volume of interest (VOI). At a resolution of 9 *μ*m, this number of slices corresponds to the region of approximately 3.5 mm in height. The next offset of 100 slices separates the trabecular ROI and cortical ROI. For the measurements, the cortical ROI was defined as 100 slices that correspond to approximately 0.9 mm. Within each ROI, the automatic selection of a specific bone was applied, and then the images were segmented using an adaptive global threshold algorithm. The quantitative analysis (CTAn, Bruker®) of the cancellous bone structure considered bone volume fraction (BV/TV), bone surface density (BS/TV), specific bone surface (BS/BV), trabecular thickness (Tb.Th), number (Tb.N) and separation (Tb.Sp) of trabeculae, structure model index (SMI), connectivity density (Conn.D), total porosity (Po.tot), and degree of anisotropy (DA).

For the cortical bone, the analysis involved the determination of average cortical thickness (Ct.Th), total cross-sectional area inside the periosteal envelope (Tt.Ar), cortical bone area (Ct.Ar), and cortical area fraction (Ct.Ar/Tt.Ar).

#### 2.6.11. Bone Histology

The histomorphometric examination of the tibia was performed in line with the 2012 update of the standardized nomenclature, symbols, and units for bone histomorphometry [65].

The left tibiae of the rats were fixed in 10% neutral buffered formalin and later decalcified in the 10% neutral buffered EDTA solution. The EDTA solution was changed once after 24 hours. The metaphyseal and epiphyseal regions of the proximal tibia were harvested, embedded in paraffin, and cut into 5 *μ*m thick slides. The slides were stained using the standard hematoxylin and eosin method and scanned using the Hamamatsu NanoZoomer 2.0 histological slide scanner and NDP.scan SQ 1.0. Finally, each sample was exported to a TIFF file.

The TIFF file was analyzed with ImageJ 1.52. Every image was briefly segmented, and the total trabecular area (B.Ar) and trabecular perimeter (B.Pm) were measured.

The bone volume to tissue volume ratio (BV/TV) was calculated as B.Ar/T.Ar. Next, the bone surface to tissue volume ratio (BS/TV) was calculated as B.Pm/T.Ar×1.2 and the BS/BV ratio as BS/TV×BV/TV. Mean trabecular thickness (Tb. Th) was calculated as 2/BS/BV.

#### 2.6.12. Mechanical Tests

**Figure 4:**
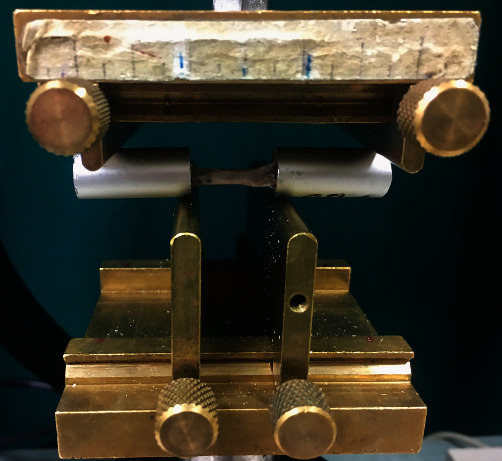
Measuring system for testing the mechanical properties of the femurs in the four-point bending test.

Mechanical properties were assessed using a four-point bending test with the MTS 858 MiniBionix machine (Eden Prairie, MN, USA) (Figure 4). In order to determine bend strength, each femoral epiphysis was fixed in aluminium alloy sleeves and embedded with the Duracryl™ Plus self-polymerised acrylic (SpofaDental, Jicin, Czech Republic). In the four-point bending test, the load was applied to the upper prisms. The spacing of the upper prisms was equal to 54 mm, and the distance between the lower support points was 24 mm. The loading speed during bending was 1 mm/min. The mechanical tests carried out in the four-point bending test led to the determination of three mechanical parameters: Young's modulus, bend strength, and bending stiffness. The values of the mechanical parameters were determined using classical formulas [66].

To calculate Young's modulus, the cross-section area of the femur sample needs to be measured. The area of each sample was determined at the point where the sample broke by finding the smallest ellipse that outlines the sample and calculating its area. The area was calculated using the measurements of the length of the ellipse's axis with the Zeiss Stereo Discovery V20, Germany stereo microscope.

#### 2.6.13. Statistical Analysis

Due to the lack of a normal distribution of the results for all examined parameters, the statistical analysis was performed with nonparametric tests (ANOVA Kruskal-Wallis and the appropriate posthoc test). The statistical analysis was performed in Statistica v.13. The significance level was *p* ≤ 0.05. All results are presented as the median (lower quartile–upper quartile).

## 3. Results

### 3.1. Body Weight

**Figure 5:**
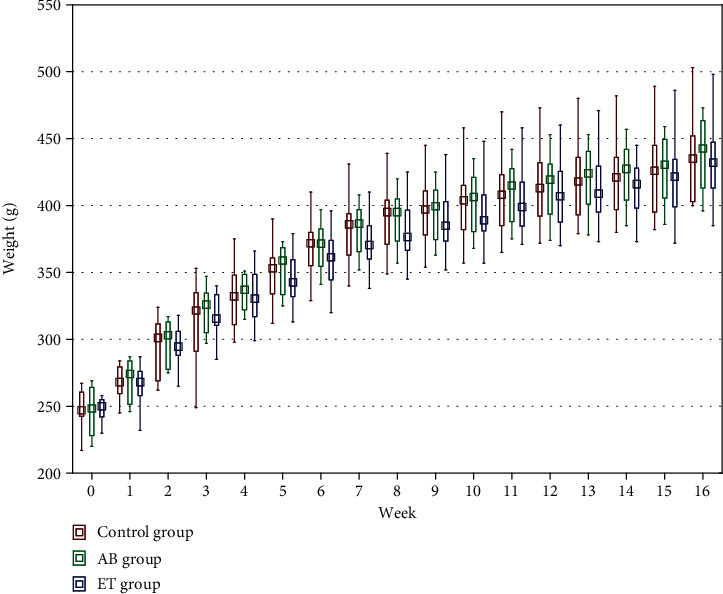
Effect of abacavir and etravirine administration on body weight. No significant difference between the analyzed groups was detected.

On the first day of the study, the control group, the AB group, and the ET group did not differ significantly in body weight. Similarly, no differences in body weight were observed between the groups during the entire study period (Figure 5).

### 3.2. Macrometric Parameters

The results are presented in Table 2. The weight of testes and the testicular index as well as the weight of the kidney and the renal index did not differ from the control group in the AB or ET group. The hepatic index was significantly higher in the ET group when compared to the control group. At the same time, the weight of the liver did not differ significantly between the AB or ET groups and the control group.

The group receiving etravirine for 16 weeks had a significantly greater tibial index, a greater diameter of the tibia, and a lower femur length than in the control group. In the group receiving abacavir at the end of the study, no differences in bone macrometric parameters were found.

### 3.3. Reproductive Hormones

The levels of reproductive hormones in serum and testicular homogenates are presented in Table 3. In the group receiving etravirine, only LH serum levels were significantly lower after 8 weeks of the study compared to the control group.

### 3.4. Minichromosome Maintenance 7 Protein

**Figure 6:**
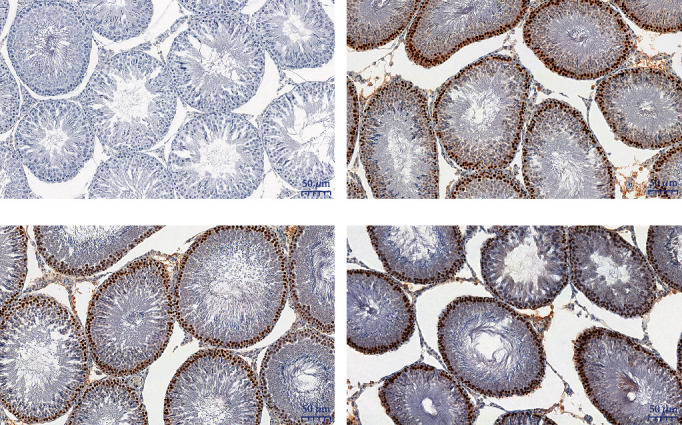
Representative slides of the expression of the MCM-7 protein in the immunohistochemical examination of testes: negative control (a), in the control group (b), the group receiving abacavir (c), and the group receiving etravirine (d). ×20 magnification.

The immunohistochemical examination revealed an increased expression of minichromosome maintenance 7 protein in the ET group compared to the control group (36.4 (35.3-37.8) % vs. 33.7 (32.8-35.5) %, *p* ≤ 0.05). No significant difference between the abacavir-receiving animals (31.7 (30.3-34.5) %) and the control group was found. The representative photos of the immunohistochemical evaluation are shown in Figure 6.

### 3.5. Semen Analysis

The results are presented in Table 4. Neither abacavir nor etravirine administration impacted total sperm count, sperm concentration, subjective motility, or sperm morphology. In the flow cytometry of semen, the percentage of cells with denatured DNA was found to be significantly greater in both study groups (AB and ET) than in the control group.

### 3.6. Redox Status

**Figure 7:**
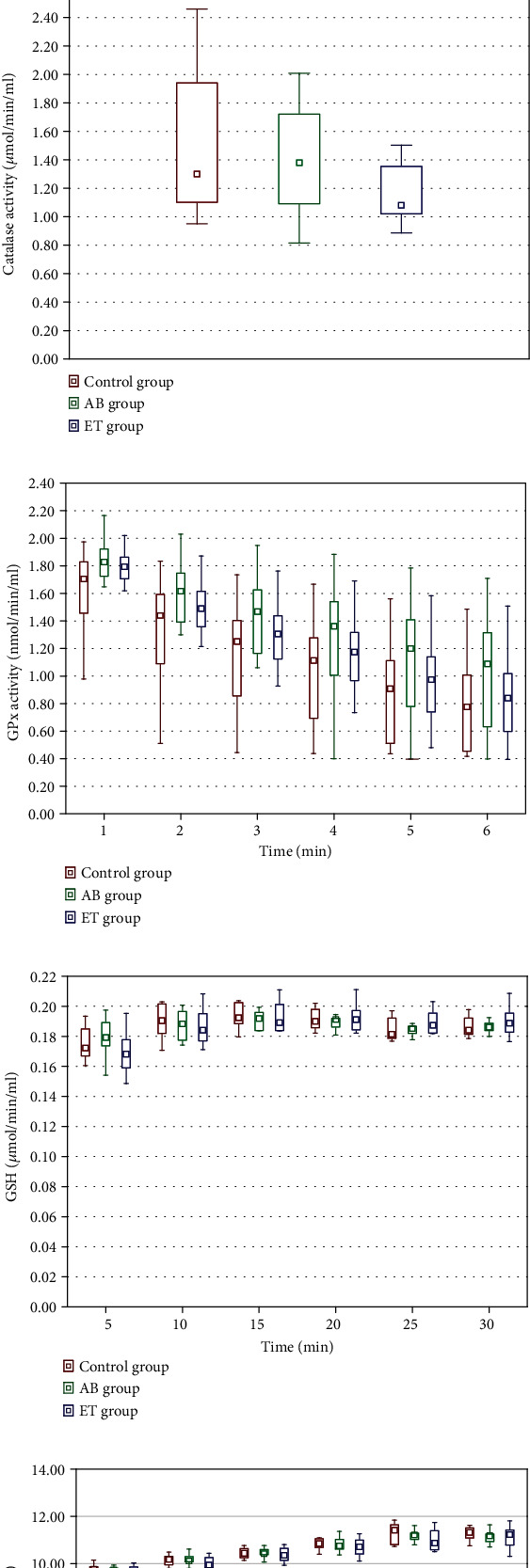
Effect of 16-week administration of abacavir and etravirine on MDA (a), superoxide dismutase activity (b), catalase activity (c), GPx activity (d), GSH (e), GSSG (f), and GSH/GSSG ratio (g) in testicular homogenates. Results presented as median (lower quartile-upper quartile); ^abacavir vs. control group, *p* ≤ 0.05; ^∗^etravirine vs. control group, *p* ≤ 0.05.

**Figure 8:**
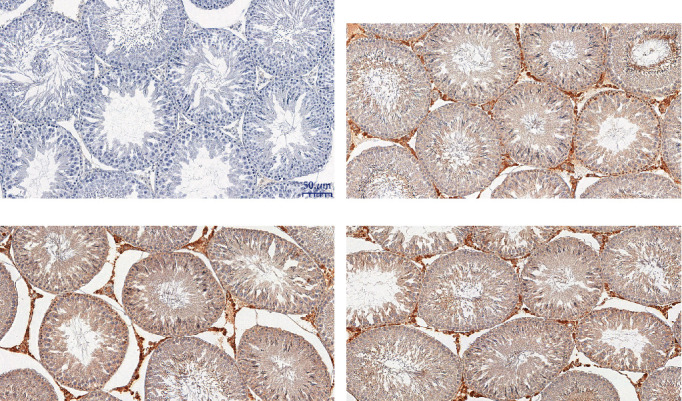
Selected cross-sections of testes (immunohistochemistry) for the glutathione expression: negative control (a), in the control group (b), the group receiving abacavir (c), and the group receiving etravirine (d). ×20 magnification.

In testicular homogenates, significantly higher SOD activity was noticed only in the AB-receiving group compared to the control rats. Etravirine did not affect SOD activity. Catalase and GPx activities and MDA and GSH levels, as well as the GSH/GSSG ratio in both AB and ET groups, were comparable to the control (Figure 7). In the immunohistochemical examination of testicular glutathione expression, no differences were revealed between the studied and control group. Representative slides are shown in Figure 8.

**Figure 9:**
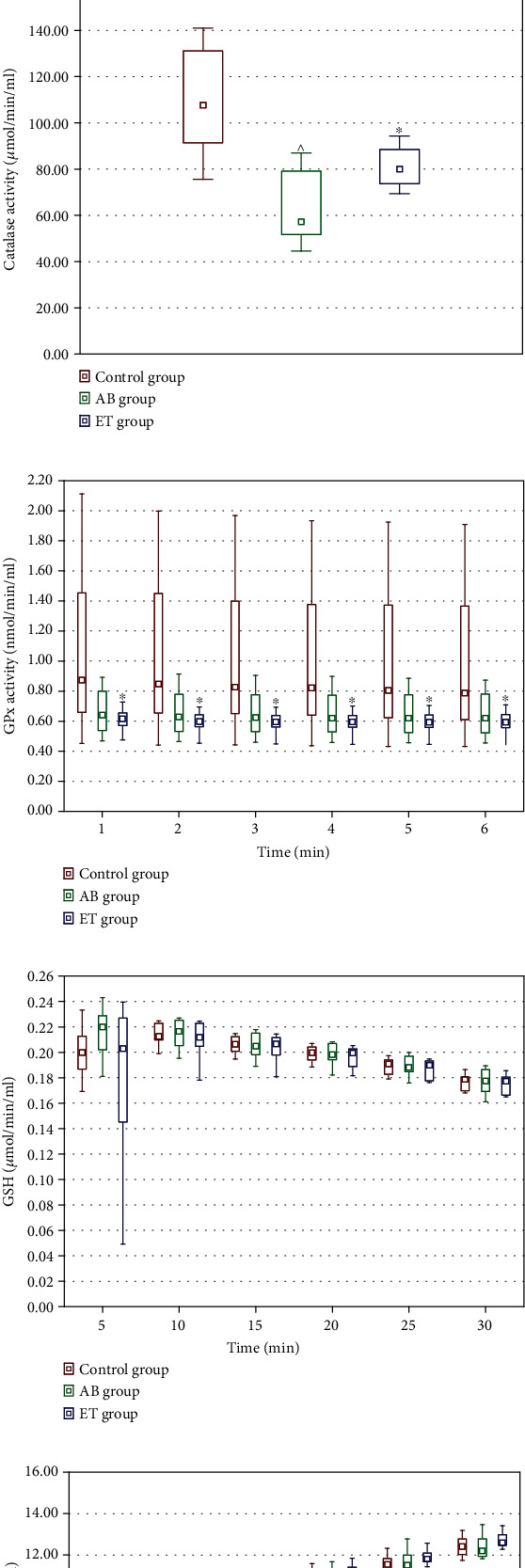
Effect of 16-week administration of abacavir and etravirine on MDA (a), superoxide dismutase activity (b), catalase activity (c), GPx activity (d), GSH (e), GSSG (f), and GSH/GSSG ratio (g) in homogenates from the liver; results presented as median (lower quartile–upper quartile); ^abacavir vs. control group, *p* ≤ 0.05; ^∗^etravirine vs. control group, *p* ≤ 0.05.

In liver homogenates, catalase activity significantly decreased in both studied groups (AB and ET) compared to the control group. Additionally, etravirine significantly decreased GPx activity at all time points under consideration (Figure 9).

**Figure 10:**
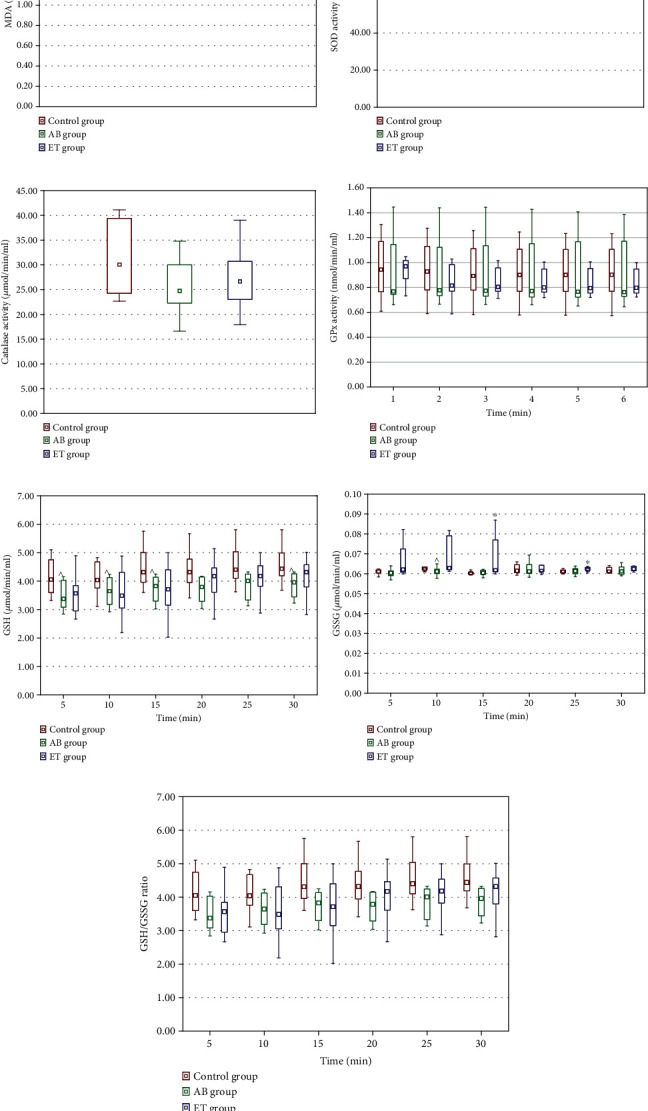
Effect of 16-week administration of abacavir and etravirine on MDA (a), superoxide dismutase activity (b), catalase activity (c), GPx activity (d), GSH (e), GSSG (f), and GSH/GSSG ratio (g) in homogenates from kidneys. Results presented as median (lower quartile–upper quartile); ^abacavir vs. control group, *p* ≤ 0.05; ^∗^etravirine vs. control group, *p* ≤ 0.05.

Abacavir decreased GSH levels in kidney homogenates, with the difference being significant at four out of the six analyzed time points. However, it did not affect the GSH/GSSG ratio. Significant differences in the GSSG concentration at single time points for AB and ET (in the 10^th^, 15^th^ and 25^th^ minutes, respectively) were noticed. In the etravirine-receiving group, SOD activity in the kidney was significantly lower (Figure 10).

### 3.7. Liver and Renal Histology

**Figure 11:**
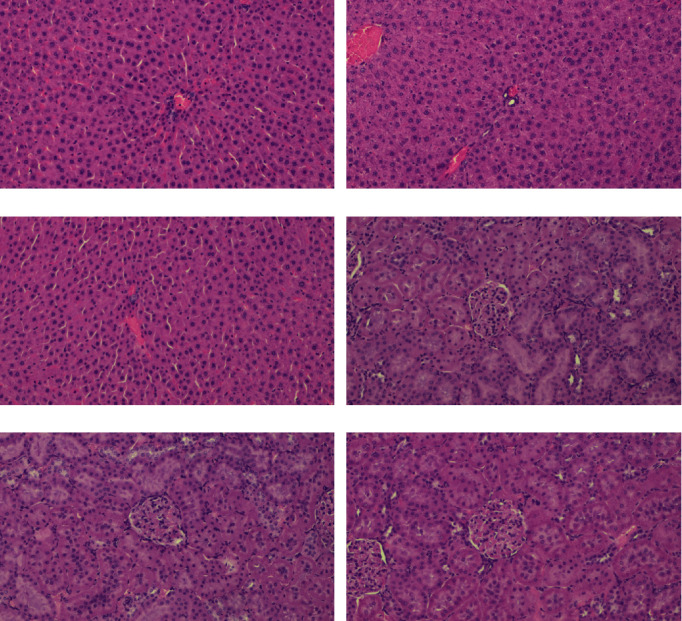
Sample images from the histopathologic examination of the liver (a–c) and kidneys (d–f) in the control group (a, d), the abacavir-receiving group (b, e), and the etravirine-receiving group (c, f).

There were no histopathological changes in the kidneys and liver in the analyzed preparations. The structure of the glomeruli and renal tubules was normal. Hepatocytes and the portal area (hepatic artery, portal vein, and bile duct) were anatomically normal. Sample images are shown in Figure 11.

### 3.8. Bone Metabolism

The results are presented in Table 5. A significant decrease in serum PINP, TRACP, and sclerostin levels between week 4 and week 16 was observed in all analyzed groups. Additionally, a decrease in serum DKK1 levels between week 4 and week 16 in the control group and in the ET group was identified. DKK1 levels were not significantly changed between week 4 and week 16 in the AB group. After 16 weeks of the study, the concentration of the Dickkopf-related protein was higher in the AB group than in the control group.

After 4 weeks of the study, the concentration of 1,25-dihydroxyvitamin D_3_ in rats receiving etravirine was lower than in the control group. After 16 weeks, creatinine levels were lower in the ET group, whereas aspartate aminotransferase activity was significantly higher in the AB group.

### 3.9. Bone Mineral Density

The results are presented in Table 6. After 16-week treatment with the analyzed drugs and/or normal saline, no statistically significant differences in tibial or femoral bone mineral density were observed between the groups.

### 3.10. Bone Histology

The histomorphometric parameters analyzed in longitudinal sections of paraffin-embedded tibiae are presented in Table 7. Bone volume fraction (BV/TV) and the bone surface/tissue volume (BS/TV) ratio were significantly higher in the abacavir-receiving group compared to the control group. No difference in trabecular thickness (Tb.Th) and BS/BV was detected between the AB and control groups. No significant impact of etravirine on tibial histomorphometric parameters was found.

### 3.11. Micro-X-Ray Computed Tomography

**Figure 12:**
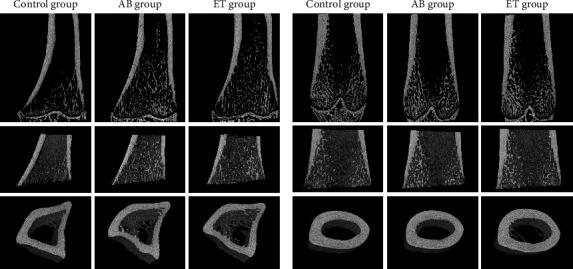
Representative mCT scans of (a) tibial bones and (b) femoral bones.

The bone morphology results vary between the tibia and femur. The results of mCT are presented in Table 8. Sample mCT scans are shown in Figure 12. No significant differences were observed for tibial and femoral Ct.Ar between the studied groups. Cortical thickness (Cr.Th) was also comparable between all three groups in both analysed localizations. The analysis of the tibial cancellous bone revealed an increase in bone volume fraction (BV/TV) for the abacavir-receiving group. Additionally, abacavir led to a higher trabecular number (Tb.N) and connectivity density (Conn.D) along with lower total porosity (Po.tot) of the tibial cancellous bone. No significant differences were observed for the femoral cancellous bone between the analyzed groups.

### 3.12. Mechanical Properties of Femurs

**Figure 13:**
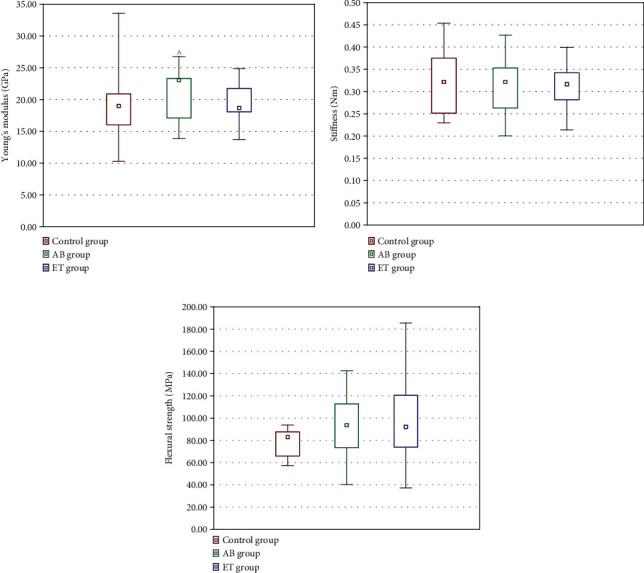
Effect of 16-week administration of abacavir and etravirine on the biomechanical properties of right femurs: Young's modulus (a), stiffness (b), and flexural strength (c). Results presented as median (lower quartile-upper quartile); ^abacavir vs. control group, *p* ≤ 0.05.

The results are given in Figure 13. In the four-point bending test, increased Young's modulus in abacavir-receiving animals was observed compared to the untreated ones. No effect of etravirine on Young's modulus was found. No statistically significant differences were found between the groups in terms of flexural strength and stiffness.

## 4. Discussion

Abnormalities in testicular and epididymal morphology, sperm morphology and motility, and oxidative stress disturbances caused by HIV infection and HAART therapy may directly affect male fertility. In animal studies, HAART therapy may influence plasma levels of sex hormones, such as testosterone, prolactin, LH, or FSH [67, 68]. HAART also caused decreased sperm count, motility, viability, and amount of sperm with normal morphology together with atrophy of seminiferous tubules and depletion of spermatogenic cells, especially of secondary spermatocytes [68, 69]. HAART had a negative effect on the frequencies and latencies of various sexual behaviour parameters in male rats and impaired their fertility with a reduced number of offspring of male HAART-receiving rodents [68, 70].

In the current study, the impact of abacavir and etravirine in male rats on reproductive toxicity was evaluated. The paper assessed the hormonal status in serum after 8 and 16 weeks of drug administration and basic morphological parameters of testes and epididymis, as well as semen morphology and viability, oxidative stress parameters, and antioxidant activity.

After 8 weeks of etravirine administration, a temporary decrease in serum LH levels was observed. After 16 weeks, serum luteinizing hormone levels were lower in the AB group than in the control group. As there was no significant difference in serum testosterone levels between all the analyzed groups, it cannot be excluded that changes in LH levels observed in this study were associated with the pulsative release of LH [71].

Oxidative stress has been reported to be one of the most important causes of male infertility, but not the only one [72]. The pathological concentration of reactive oxygen species (ROS) leads to excessive lipid peroxidation, DNA damage, and cellular apoptosis [73]. In the male reproductive system, the proper balance between oxidative stress and antioxidant systems is particularly important because ROS in low concentrations are necessary for physiological sperm functions such as spermatogenesis, capacitation, acrosome reaction, motility of sperm flagella, or fertilization. Excessive oxidative stress resulting from imbalances between antioxidants and free radicals may be toxic and account for infertility. Plasma membranes in sperm are rich in unsaturated fatty acids, making them very susceptible to peroxidation and damage.

Moreover, spermatozoa contain only little cytoplasm, resulting in insufficient cytoplasmic antioxidant defense [74, 75]. The cells are protected from oxidative stress by an enzymatic (CAT, SOD, GPx) and nonenzymatic (GSH) antioxidant system [72]. SOD converts superoxide anion (O_2_^−^) to hydrogen peroxide (H_2_O_2_) and prevents the formation of highly reactive hydroxyl radicals. Hydrogen peroxide is further directly converted to H_2_O by catalase or by glutathione peroxidase using the reduced glutathione (GSH) molecule, which is converted to the oxidized glutathione form (GSSG) [76]. MDA is a stable end product of the formation of free radicals and is the main biomarker for oxidative stress analysis and monitoring in various tissues, including testes [75].

Several in vitro studies suggest that antiretroviral drugs may generate ROS. It was found that various nucleoside reverse transcriptase inhibitors (NRTIs) impair the mitochondrial function in HepG2 cells and alter oxidative stress parameters causing, e.g., an increase in MDA levels (zidovudine and tenofovir) and a decrease in the GSH concentration (stavudine) [77]. Efavirenz, the first-generation of nonnucleoside reverse transcriptase inhibitors (NNRTIs), was also found to generate oxidative stress in endothelial cell lines [78, 79]. ROS generation is largely due to interference with mitochondrial function, altered replication of DNA in mitochondria, and inhibition of oxidative phosphorylation processes [80].

In the AB and ET groups, increased DNA instability expressed by an elevated percentage of spermatozoa with denatured DNA was found. In the study of testicular homogenates, abacavir was the only drug to significantly increased activity of SOD, which is one of the first in the enzymatic antioxidant pathway. No other changes in prooxidant (MDA) or enzymatic/nonenzymatic antioxidants were noticed in testes, suggesting that the described changes in DNA instability in spermatozoa are not the direct consequence of oxidative stress generation. It must, however, be stated that oxidative stress parameters in the seminal plasma were not assessed in this study and not affected the redox state in testes, not definitely excluding imbalances between pro- and antioxidants in semen.

Zini et al. reported that male infertility is associated with poor sperm DNA stability [81]. They also noted that if assisted conception is performed, fertilization with DNA-damaged spermatozoa may increase the risk of genetic diseases in children. The doubled percentage of spermatozoa with denatured DNA in abacavir- and etravirine-receiving rats supports the hypothesis that these drugs may impair male fertility due to increasing DNA instability, which should be confirmed in further studies, e.g., one focusing on the fertilization rate.

More pronounced changes in oxidative stress parameters were detected in the liver and kidneys. Etravirine significantly affects SOD activity in kidneys as well as catalase and GPx activities in the liver, suggesting the possible negative impact on the state of antioxidants; still, this does not affect MDA levels—the main prooxidant end product. Similarly, abacavir decreases catalase activity in the liver and GSH levels in rat kidneys without affecting the MDA concentration in both tissues. Both drugs are metabolized in the liver, but different metabolic pathways are important. Abacavir metabolism depends mainly on alcohol dehydrogenase and glucuronidation [82]. Etravirine is metabolized by CYP3A and CYP2C enzymes and later undergoes glucuronidation [83]. It seems that the 16-week abacavir administration affects the liver to a greater extent than etravirine administration, which was also reflected in significantly elevated AST activity in the AB-receiving group and a higher liver index. In the histological assessment, no pathological changes in the structure of livers obtained from both AB and ET groups were described, suggesting that AB causes functional rather than morphological injury.

Despite some alterations in the redox state in kidneys, the morphology of AB or ET administration was not affected, as shown in the histological evaluation. Moreover, serum creatinine levels were not significantly changed in the AB group and even were lowered significantly in the ET group, meaning that both drugs are rather not nephrotoxic. The detected mild decrease in creatinine levels in the ET-receiving animals had no clinical significance as the obtained results were within the normal limits for rats for an enzymatic method of plasma creatinine determination [84].

The lack of significant changes in SOD, CAT, and GPx activities and GSH and MDA concentrations in testes between the groups, but the presence of significant differences in the oxide–redox state between the studied and control groups in the liver and kidney, may result from drug pharmacokinetics. Drug concentration in plasma and in other body fluids and tissues depends on absorption, biotransformation, distribution, and excretion [85]. Following absorption from the gastrointestinal tract, drugs are transported to the liver by portal circulation to reach general circulation and are distributed to various tissues [86]. However, drug concentrations in tissues may vary greatly and depend on the drug molecular size, degree of protein binding, membrane permeability, presence of specific blood-tissue barriers, and tissue blood flow [87]. The delivery of drugs to the organs is mainly determined by the tissue blood flow. The accumulation of drugs in highly perfused tissues is greater than in tissues with low blood perfusion [85]. It is estimated that the blood flow in the renal cortex is about 700 ml/min/100 g of tissue, in the liver about 100-130 ml/min/100 g of tissue, and in testes only about 9-12 ml/min/100 g of tissue [88–90]. Further, the liver is the main place for drug metabolism, possibly resulting in reactive toxic metabolites [82, 91]. In kidneys, being the most important excreting organ, primary urine is concentrated and, thus, kidneys are exposed to high concentrations of drugs and their metabolites [92]. Studies on abacavir pharmacokinetics in mice that were given a single oral dose of 10 mg/kg confirm that after 15 minutes, the highest concentrations were found in the gallbladder, digestive tract, and kidneys, followed by a decrease below the detectable limit within 16 hours. In pregnant rats given the same oral dose, a high abacavir concentration after 6 hours was found in kidneys and the liver, whereas high abacavir levels in the liver were still present after 48 hours [93]. The data obtained from animal pharmacokinetic studies reveal that high etravirine levels were found in the liver and renal cortex compared to the lowest concentrations determined in, e.g., the seminal vesicle [94].

The balance between cell proliferation and apoptosis is important for the maintenance of male fertility [95]. In this study, etravirine increased the MCM-7 protein (proliferation marker) expression in testes, pointing to the imbalance between proliferation and apoptosis. The consequence of increased proliferation may include the formation of functionally immature Sertoli cells [96].

No effect of abacavir on femoral and tibial BMD was demonstrated in this study. These findings are in line with the observations reported by Stelbrink et al. [97]. Their study revealed that a BMD loss was significantly greater (about 6%) in the tenofovir-emtricitabine group than in the abacavir-lamivudine group [97]. Negredo et al. also reported that introducing abacavir instead of tenofovir into therapy exerted beneficial BMD effects [98]. The histomorphometric analysis of tibial sections yields significantly higher bone volume fraction (BV/TV) and bone surface density (BS/TV) in the abacavir-receiving animals. In microcomputed tomography of the tibial trabecular bone, an increased trabecular number (Tb.N) and connectivity density (Conn.D) associated with lower total porosity in the AB group were also identified. All other microarchitectural parameters determined by mCT analysis were comparable in all three analyzed groups. The four-point bending test revealed increased Young's modulus in the AB group, indicating increased tensile stiffness of the femoral bone in the abacavir-receiving animals. Stiffness and flexural strength were comparable between the analyzed groups. Higher Tb.N and Conn.D may be attributed to the fact that abacavir was reported to activate adenosine A_2_ receptors (A_2_R) [99]. Medeiro et al. demonstrated that the A_2_R agonist inhibited osteoclast differentiation [100]. Based on these reports, it may be hypothesized that abacavir, through the activation of A_2_R, may shift the balance between bone resorption and bone formation towards osteogenesis. However, in the in vitro study using the osteosarcoma Saos-2 cell line as a model, it was observed that abacavir inhibited osteoblast differentiation and decreased the ability of the cells to form calcium deposits in the extracellular matrix [32].

Further studies are needed to explain the effect of abacavir on bone metabolism. Bone strength depends not only on bone mineralisation but also on bone geometry and the shape of bones, the microarchitecture of trabecular bones, turnover, and collagen in the extracellular matrix. Even though Esposito et al. reported that abacavir impairs the mineralisation and synthesis of type I collagen [32], it did not decrease the mechanical resistance of femoral bones in this study. It is suspected that the increased number and trabeculae and elevated connectivity density detected in mCT counteracted the probable unfavorable impact of abacavir-induced mineralization inhibition on bone mechanical properties.

In this study, serum levels of bone turnover markers were measured twice—in week 4 and in week 16. In week 4, there was no difference in serum levels of PINP, TRACP, sclerostin, and Dickkopf-related protein 1 (DKK1) between the groups under analysis. In week 16, serum levels of PINP, TRACP, and sclerostin remained comparable between all groups. Serum DKK1 levels were elevated in the AB group compared to the control group. No difference in serum DKK1 levels between the control group and the ET group was detected. Lower serum levels of PINP and TRACP observed in the study between week 4 and week 16 resulted from an inhibition of bone turnover associated with growth slowing (Table 6, Figure 3). Sclerostin and DKK1 are both Wnt antagonists. The increase in DKK1 levels in the AB group may lead to slower bone formation and growth retardation if it should persist for a longer time. In the mice model, increased DKK1 levels were associated with osteopenia [101]. However, Ueland et al. found that femoral and spine BMD in postmenopausal women were positively correlated with cortical and trabecular DKK1 levels, respectively. Cortical DKK1 was also positively correlated with volumetric bone density and biomechanical strength [102]. This study also points to better bone parameters in the abacavir-receiving group despite increased serum DKK1 levels, which requires further detailed investigations and measurements of sclerostin or DKK1 levels in bone biopsies.

In this study, femoral and tibial BMDs were comparable between the ET and control groups. Additionally, the present study did not demonstrate any effect of etravirine on bone microarchitecture in neither in bone histomorphometry nor bone mCT examination. These findings are consistent with the results presented by other authors [103, 104], who found no negative impact of the therapy with raltegravir plus etravirine and ritonavir-boosted darunavir plus etravirine on bones. In the present study, etravirine was found to have no effect on bone mechanical properties, supporting the hypothesis that it does not exert a harmful effect on bones in growing rats.

In the study using the osteosarcoma Saos-2 cell line as a model, it was observed that the presence of etravirine in the culture medium increased the number of calcium deposits, pointing to an increase in final osteoblast differentiation and an increase in bone formation [28]. In this study, in the group receiving etravirine for 16 weeks, a higher tibial index and greater mid-tibial diameter were determined. This suggests an increase in bone formation, which is consistent with the in vitro results [32]. In rats, bone turnover is more rapid in the tibia than in the femur, which explains why changes in the tibial index without changes in the femoral index were found in the present study. Even though the tibial index was higher in the ET group than in the control one, PINP levels were comparable between both groups. PINP is a bone formation marker, with its levels being correlated with the formation of matrix collagen in bones. Considering the results reported by Esposito et al. [32] and the results presented in this study, it may be suspected that etravirine increases calcium deposit formation but without promoting the synthesis of collagen in the bone matrix. However, it cannot be excluded that rapid bone turnover in growing rats masked the effect of etravirine on collagen formation. The lack of a statistically significant difference between the ET group and the control group in terms of serum PINP levels (bone formation marker) may result from significant and rapid bone turnover in the growing period, making observations difficult. What is more, concentrations of bone turnover markers in serum reflect the current intensity of bone formation and resorption, but do not confirm the intensity of these processes in a longer period [105].

In the 4^th^ week of this study, a significant decrease in serum 1,25-dihydroxyvitamin D_3_ levels was measured in the etravirine-receiving group. Decreased vitamin D concentrations and the impairment of vitamin D metabolic pathways by antiretroviral drugs (especially HIV protease inhibitors and nonnucleoside reverse transcriptase inhibitors) were found by other authors, which were confirmed by both in vitro and in vivo studies [106, 107]. Efavirenz, belonging to the same group as etravirine, was found to increase 25-hydroxyvitamin D catabolism, resulting in increased levels of inactive metabolites [106, 107]. To date, no studies have been conducted to assess the effect of etravirine on BMD. Long-term vitamin D deficiency could lead to bone mineralization disorders and a decrease in bone mineral density. This study assessed vitamin D levels twice—in week 4 and week 16 of the study. It is impossible to predict how long vitamin D deficiency lasted after one random evaluation during the study. Despite transiently decreased vitamin D levels, no differences in bone mineral density, histomorphometric parameters, or mechanical properties of bones were demonstrated between the ET-receiving and control groups. This may be explained by further positive impact of etravirine on bone formation.

## 5. Conclusions

Abacavir and etravirine doubled the percentage of sperms with denatured DNA, suggesting decreased fertility in animals receiving these drugs. The results of this study indicate that etravirine may decrease serum vitamin D concentration, which dictates the need to monitor vitamin D levels in etravirine-receiving children. However, no harmful effect of etravirine on bones in growing rats was identified. Abacavir was found to improve bone microarchitecture in growing rats but may affect the liver function.

## Figures and Tables

**Figure 2:**
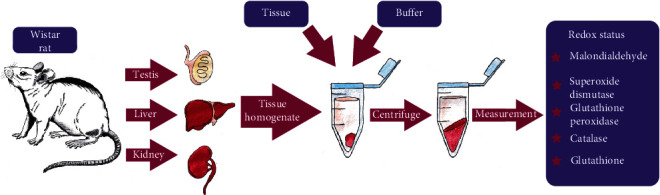
General diagram of tissue homogenate preparation for redox state measurements.

**Table 1:** Conversion of a single dose of the tested anti-retroviral drugs from human to rats.

Drug	Human dose	Corresponding rat dose
Abacavir	8 mg/kg-10 mg/kg	49.6 mg/kg-62 mg/kg
Etravirine	5.7 mg/kg-6.7 mg/kg	35.3 mg/kg-41.5 mg/kg

**Table 2:** Effect of 16-week administration of abacavir and etravirine on macrometric parameters (AB group: group receiving abacavir 60 mg/kg for 16 weeks; ET group: group receiving etravirine 40 mg/kg for 16 weeks). Results presented as median (lower quartile–upper quartile); ^AB group vs. control group, *p* ≤ 0.05; ^∗^ET group vs. control group, *p* ≤ 0.05.

	Control group	AB group	ET group
Weight of testis [g]	1.736 (1.459-1.895)	1.742 (1.644-1.854)	1.706 (1.570-1.797)
Testicular index [%]	0.790 (0.677-0.876)	0.775 (0.739-0.857)	0.781 (0.679-0.831)
Weight of liver [g]	10.49 (9.96-11.03)	11.13 (10.75-11.93)	11.21 (10.92-12.00)
Hepatic index [%]	2.405 (2.310-2.503)	2.598 (2.500-2.664)	2.680 (2.469-2.788)^∗^
Weight of kidney [g]	1.275 (1.147-1.425)	1.311 (1.192-1.398)	1.264 (1.207-1.357)
Renal index [%]	0.578 (0.532-0.618)	0.591 (0.571-0.605)	0.599 (0.537-0.645)
Tibia weight [g]	0.787 (0.753-0.841)	0.827 (0.792-0.840)	0.830 (0.820-0.873)
Tibial index [%]	0.183 (0.174-0.192)	0.183 (0.178-0.197)	0.195 (0.184-0.210)^∗^
Tibia length [mm]	41.73 (41.38-42.45)	41.94 (41.74-42.38)	41.82 (41.27-42.52)
Mid-tibial diameter [mm]	2.47 (2.43-2.52)	2.54 (2.41-2.67)	2.61 (2.55-2.66)^∗^
Femur weight [g]	1.151 (1.131-1.173)	1.151 (1.095-1.178)	1.150 (1.096-1.189)
Femoral index [%]	0.265 (0.253-0.271)	0.255 (0.247-0.273)	0.271 (0.255-0.281)
Femur length [mm]	38.24 (37.65-38.62)	37.88 (37.72-38.65)	37.64 (37.34-38.18)^∗^
Mid-femoral diameter [mm]	3.485 (3.36-3.53)	3.505 (3.435-3.630)	3.59 (3.51-3.68)

**Table 3:** Effect of 16-week administration of abacavir and etravirine on levels of reproductive hormones in serum and in testicular homogenates (AB group: group receiving abacavir 60 mg/kg for 16 weeks; ET group: group receiving etravirine 40 mg/kg for 16 weeks; LH: luteinising hormone; FSH: follicle-stimulating hormone; SHBG: sex hormone-binding globulin; TSH: thyroid-stimulating hormone. Results presented as median (lower quartile–upper quartile); ^AB group vs. control group, *p* ≤ 0.05; ^∗^ET group vs. control group, *p* ≤ 0.05).

		Control group	AB group	ET group
Week 8 serum	LH [mIU/ml]	4.93 (4.39-5.64)	4.60 (3.52-5.38)	3.99 (3.83-4.48)^∗^
	FSH [mIU/ml]	2.09 (1.95-2.31)	2.05 (1.83-2.23)	2.25 (2.13-2.36)
	Testosterone [ng/l]	104.1 (93.5-109.0)	98.1 (88.4-104.6)	105.7 (94.0-112.7)
	Estradiol [ng/l]	33.6 (32.6-36.3)	33.8 (32.2-36.2)	33.3 (31.4-35.6)
	SHBG [ng/ml]	1.52 (1.26-1.67)	1.26 (1.09-1.67)	1.31 (1.09-1.52)

Week 16 serum	LH [mIU/ml]	4.0 (3.54-4.42)	3.30 (3.13-3.40)	4.12 (3.33-4.25)
	FSH [mIU/ml]	1.94 (1.73-2.08)	1.89 (1.62-2.15)	1.92 (1.51-2.22)
	Testosterone [ng/l]	99.8 (91.0-113.3)	97.6 (88.7-107.3)	100.3 (91.7-104.9)
	Estradiol [ng/l]	31.5 (28.0-35.8)	32.0 (29.5-33.1)	33.0 (30.7-34.6)
	Inhibin B [ng/l]	27.2 (17.7-28.8)	26.8 (22.2-33.7)	26.3 (21.7-29.2)
	SHBG [ng/ml]	1.35 (1.26-1.61)	1.39 (1.22-1.64)	1.24 (1.13-1.51)
	Prolactin [ng/ml]	8.18 (5.82-9.23)	7.62 (6.13-8.07)	6.77 (6.21-8.41)
	TSH [mIU/ml]	1.68 (1.42-2.06)	1.96 (1.56-2.05)	1.76 (1.57-1.95)

Week 16 testicular homogenates	Testosterone [ng/l]	71.1 (68.8-74.1)	76.6 (72.4-84.1)	82.9 (72.7-94.1)
	Estradiol [ng/l]	173.9 (169.0-185.0)	194.4 (164.1-208.7)	192.6 (172.0-205.4)
	Inhibin B [ng/l]	54.6 (43.5-57.1)	59.5 (50.7-64.8)	52.7 (47.0-58.4)

**Table 4:** Effect of 16-week administration of abacavir and etravirine on semen parameters (AB group: group receiving abacavir 60 mg/kg for 16 weeks; ET group: group receiving etravirine 40 mg/kg for 16 weeks; DFI: DNA fragmentation index; HDS: high DNA stainability. Results presented as median (lower quartile–upper quartile); ^abacavir vs. control group, *p* ≤ 0.05; ^∗^etravirine vs. control group, *p* ≤ 0.05).

		Control group	AB group	ET group
Total sperm count [mln]		58.9 (40.7-87.6)	64.2 (54.4-84.1)	63.8 (42.8-75.6)

Sperm concentration [mln/ml]		73.7 (50.8-109.5)	80.3 (68.0-102.1)	79.8 (53.5-94.6)

Subjective motility [%]		57.5 (55-65)	62.5 (50-65)	62.5 (52.5-65)

Sperm morphology	Morphologically normal [%]	93.0 (87.2-93.2)	88.6 (87.2-92.2)	92.9 (83.6-96.1)
	Proximal droplet [%]	0 (0-0)	0 (0-0)	0 (0-0)
	Distal droplet [%]	0.75 (0-0.75)	0.62 (0.25-1.50)	0.5 (0.37-0.75)
	Head abnormalities [%]	0 (0-0)	0 (0-0.25)	0 (0-0.37)
	Detached head [%]	2.5 (2-3)	2.87 (2.5-3.5)	1.87 (1.0-4.37)
	Acrosome abnormalities [%]	0 (0-0)	0 (0-0)	0 (0-0)
	Midpiece defects [%]	2.25 (1-6.2)	5.5 (2.2-6.2)	2.12 (0.87-7.5)
	“Dag-like” defect [%]	0 (0-0)	0 (0-0)	0 (0-0)
	Bent tail [%]	2 (1-2.7)	1.6 (1.2-3.0)	1.1 (0.37-3.0)
	Coiled tail [%]	0 (0-0)	0 (0-0)	0 (0-0)

Plasma membrane and acrosome integrity	Live cells with intact acrosome [%]	39.7 (33.9-45.2)	42.3 (32.3-44.9)	44.5 (40.8-50.2)
	Live cells with ruptured acrosome [%]	0.23 (0.1-0.23)	0.17 (0.12-0.31)	0.15 (0.12-0.24)
	Dead cells with intact acrosome [%]	58.9 (52.3-63.7)	56.0 (53.5-66.5)	54.0 (47.9-57.2)
	Dead cells with ruptured acrosome [%]	2.09 (1.28-2.19)	1.57 (1.25-2.05)	1.60 (0.87-2.34)

Mitochondrial activity	High mitochondrial activity [%]	6.54 (5.68-7.85)	3.20 (1.71-5.17)	3.25 (1.94-4.72)
	Low mitochondrial activity [%]	93.5 (92.1-94.3)	96.8 (94.8-98.3)	96.7 (95.3-98.0)

Lipid peroxidation	Live without LPO [%]	23.3 (20.0-25.7)	24.7 (22.5-27.3)	24.7 (22.5-27.3)
	Live with LPO [%]	0.06 (0.03-0.21)	0.05 (0.03-0.16)	0.05 (0.03-0.16)
	Dead without LPO [%]	75.7 (73.7-78.2)	74.5 (72.1-76.4)	74.5 (72.1-76.4)
	Dead with LPO [%]	0.79 (0.57-0.82)	0.68 (0.51-1.15)	0.68 (0.51-1.15)

Apoptosis and necrosis	Live cells [%]	42.6 (31.0-44.2)	48.8 (45.7-51.9)	54.3 (41.0-60.3)
	Dead cells [%]	55.1 (54.5-65.5)	48.5 (45.4-51.0)	43.3 (38.4-55.2)
	Apoptotic and necrotic cells [%]	1.64 (1.28-2.09)	1.85 (1.72-2.27)	2.20 (1.33-4.56)
	Apoptotic cells [%]	0.61 (0.07-0.82)	0.75 (0.12-1.25)	0.06 (0.02-0.12)

Chromatin status	DFI [%]	0.85 (0.61-1.00)	1.56 (1.18-2.00)^	1.41 (1.17 − 2.03)∗
	HDS [%]	18.5 (10.5-20.5)	8.0 (6.5-12.1)	12.1 (11.6-18.6)

**Table 5:** Effect of abacavir and etravirine administration on serum parameters after 4 and 16 weeks (AB group: group receiving abacavir 60 mg/kg for 16 weeks; ET group: group receiving etravirine 40 mg/kg for 16 weeks; PINP: N-terminal propeptide of type I procollagen; TRACP: tartrate-resistant acid phosphatase from 5b; DKK1: Dickkopf-related protein 1; OPG: osteoprotegerin; 25-OH-D: 25-hydroxyvitamin D. Results presented as median (lower quartile-upper quartile); ^AB group vs. control group, *p* ≤ 0.05; ^∗^ET group vs. control group, *p* ≤ 0.05).

		Control group	AB group	ET group
Week 4	PINP [ng/ml]	14.6 (10.9-18.9)	18.1 (11.9-23.0)	12.4 (7.0-17.6)
	TRACP [U/l]	2.4 (1.8-2.8)	2.2 (2.0-2.7)	2.6 (1.9-2.9)
	Sclerostin [pg/ml]	1037.7 (983.6-1336.8)	1101.0 (1008.6-1193.0)	1110.6 (982.8-1182.1)
	DKK1 [ng/ml]	3.9 (3.5-7.7)	5.3 (3.2-6.7)	5.2 (4.4-7.8)
	OPG [pg/ml]	165.5 (154.0-169.5)	150.1 (132.9-163.4)	123.9 (98.7-167.4)
	1,25-dihydroxyvitamin D_3_ [nmol/l]	7.9 (6.0-8.2)	6.9 (6.5-7.6)	6.8 (5.4 − 7.6)^∗^

Week 16	PINP [ng/ml]	0.8 (0.5-1.1)	0.7 (0.6-0.8)	0.7 (0.6-0.9)
	TRACP [U/l]	1.3 (0.9-1.5)	1.1 (1.0-1.3)	1.1 (1.0-1.3)
	Sclerostin [pg/ml]	836.3 (726.6-1047.0)	830.7 (559.4-884.6)	859.7 (774.5-1010.1)
	DKK1 [ng/ml]	3.0 (1.9-5.3)	5.4 (3.2-12.5)^	2.7 (2.0-5.5)
	OPG [pg/ml]	79.7 (70.0-96.1)	86.4 (58.9-113.9)	102.9 (73.4-124.5)
	25-OH-D [nmol/l]	2.0 (1.7-2.1)	2.0 (1.8-2.2)	2.0 (1.8-2.2)
	1,25-dihydroxyvitamin D_3_ [nmol/l]	6.3 (5.8-9.3)	6.8 (4.5-7.3)	6.6 (5.2-8.1)
	Parathormone [pg/ml]	42.0 (33.7-53.3)	47.0 (41.9-51.5)	49.2 (45.6-52.1)
	Total calcium [mg/dl]	9.3 (9.0-9.6)	9.3 (8.9-9.5)	9.1 (9.0-9.3)
	Inorganic phosphorus [mg/dl]	6.0 (5.0-7.4)	6.2 (5.7-7.6)	5.2 (5.0-6.6)
	Aspartate aminotransferase [U/l]	0.33 (0.25-0.50)	0.51 (0.48-0.75)^	0.39 (0.34-0.55)
	Creatinine [mg/dl]	0.33 (0.29-0.38)	0.33 (0.28-0.35)	0.29 (0.26-0.33)^∗^

**Table 6:** Effect of 16-week administration of abacavir and etravirine on the bone mineral density (AB group: group receiving abacavir 60 mg/kg for 16 weeks; ET group: group receiving etravirine 40 mg/kg for 16 weeks. Results presented as median (lower quartile–upper quartile).

	Control group	AB group	ET group
Tibial BMD [g/cm^2^]	0.223 (0.220-0.234)	0.228 (0.224-0.237)	0.231 (0.226-0.234)
Femoral BMD [g/cm^2^]	0.276 (0.265-0.284)	0.272 (0.265-0.279)	0.276 (0.271-0.283)

**Table 7:** Effect of 16-week administration of abacavir and etravirine on the tibia assessed using histopathological examination (AB group: group receiving abacavir 60 mg/kg for 16 weeks; ET group: group receiving etravirine 40 mg/kg for 16 weeks; BV/TV: bone volume/tissue volume; BS/TV: bone surface/tissue volume; BS/BV: bone surface/bone volume ratio; Tb.Th: trabecular thickness. Results presented as median (lower quartile–upper quartile); ^AB group vs. control group, *p* ≤ 0.05).

	Control group	AB group	ET group
BV/TV [%]	18.3 (12.6-21.6)	24.6 (23.3-26.4)^	17.2 (14.4-19.8)
BS/TV [mm^2^/mm^3^]	7.61 (7.17-9.49)	9.93 (9.68-10.7)^	8.41 (7.09-9.54)
BS/BV [mm^2^/mm^3^]	47.5 (36.2-53.4)	40.8 (35.7-46.3)	49.1 (41.4-52.6)
Tb.Th [mm]	0.042 (0.037-0.055)	0.049 (0.043-0.0056)	0.041 (0.038-0.048)

**Table 8:** Effect of 16-week administration of abacavir and etravirine on the cancellous and cortical bone of the tibia and femur assessed using micro-X-ray computed tomography (AB group: group receiving abacavir 60 mg/kg for 16 weeks; ET group: group receiving etravirine 40 mg/kg for 16 weeks; BV/TV: bone volume fraction; BS/TV: bone surface density; BS/BV: specific bone surface; Tb.Th: trabecular thickness; Tb.N: trabecular number; Tb.Sp: trabecular separation; SMI: structure model index; Conn.D: connectivity density; Po.tot: total porosity; DA: degree of anisotropy; Ct.Th: average cortical thickness; Tt.Ar: total cross-sectional area inside the periosteal envelope; Ct.Ar: cortical bone area; Ct.Ar/Tt.Ar: cortical area fraction. Results presented as median (lower quartile–upper quartile); ^AB group vs. control group, *p* ≤ 0.05).

			Control group	AB group	ET group
Tibia	Cancellous bone	BV/TV [%]	13.2 (11.6-14.3)	14.7 (12.7-17.9)**^**	13.8 (12.7-15.9)
		BS/TV [mm^2^/mm^3^]	6.1 (4.6-6.6)	6.7 (5.6-7.7)	6.5 (5.8-6.8)
		BS/BV [mm^2^/mm^3^]	45.0 (43.0-47.9)	43.8 (42.7-46.4)	44.9 (42.6-47.2)
		Tb.Th [mm]	0.083 (0.080-0.087)	0.084 (0.081-0.086)	0.083 (0.080-0.086)
		Tb.N [1/mm]	1.64 (1.23-1.74)	1.80 (1.49-2.11)^	1.75 (1.54-1.87)
		Tb.Sp [mm]	0.52 (0.49-0.77)	0.44 (0.38-0.58)	0.44 (0.41-0.56)
		SMI [-]	2.15 (2.04-2.23)	2.07 (1.95-2.16)	2.01 (1.95-2.22)
		Conn.D [1/mm^3^]	41.4 (27.4-45.5)	46.3 (35.5-59.8)^	44.5 (38.7-48.2)
		Po.tot [%]	86.8 (85.6-88.3)	85.2 (82.1-87.3)^	86.2 (84.1-87.3)
		DA [-]	1.70 (1.54-1.76)	1.65 (1.62-1.75)	1.68 (1.59-1.76)

Tibia	Cortical bone	Ct.Th [mm]	0.62 (0.61-0.65)	0.63 (0.60-0.64)	0.63 (0.62-0.65)
		Tt.Ar [mm^2^]	48.3 (47.8-49.1)	50.7 (48.7-52.4)	51.2 (49.2-53.1)
		Ct.Ar [mm^2^]	63.2 (61.3-65.8)	66.2 (62.6-69.4)	64.4 (63.0-69.6)
		Ct.Ar/tt.Ar [%]	1.29 (1.26-1.31)	1.29 (1.26-1.33)	1.28 (1.25-1.36)

Femur	Cancellous bone	BV/TV [%]	18.3 (15.7-19.0)	18.2 (16.4-20.4)	18.7 (16.7-20.3)
		BS/TV [mm^2^/mm^3^]	7.85 (6.14-8.12)	7.37 (7.11-8.73)	7.83 (7.48-8.46)
		BS/BV [mm^2^/mm^3^]	42.5 (41.0-44.1)	42.7 (41.8-43.3)	42.9 (41.7-44.8)
		Tb.Th [mm]	0.086 (0.084-0.089)	0.085 (0.083-0.088)	0.085 (0.083-0.087)
		Tb.N [1/mm]	2.16 (1.71-2.22)	2.04 (1.93-2.40)	2.16 (2.02-2.31)
		Tb.Sp [mm]	0.53 (0.49-0.75)	0.56 (0.42-0.68)	0.52 (0.45-0.61)
		SMI [-]	1.76 (1.72-1.81)	1.71 (1.61-1.77)	1.69 (1.65-1.84)
		Conn.D [1/mm^3^]	75.4 (52.9-80.6)	73.5 (69.6-86.9)	79.5 (71.2-84.3)
		Po.tot [%]	81.7 (81.0-84.3)	81.8 (79.5-83.6)	81.3 (79.7-83.3)
		DA [-]	1.23 (1.22-1.26)	1.21 (1.17-1.25)	1.24 (1.21-1.25)

Femur	Cortical bone	Ct.Th [mm]	0.74 (0.73-0.76)	0.73 (0.70-0.75)	0.72 (0.71-0.74)
		Tt.Ar [mm^2^]	47.5 (45.2-48.7)	47.9 (47.0-49.4)	48.6 (47.0-51.0)
		Ct.Ar [mm^2^]	56.8 (55.3-57.6)	57.5 (55.8-62.2)	60.0 (56.0-63.4)
		Ct.Ar/tt.Ar [%]	1.20 (1.17-1.22)	1.21 (1.18-1.26)	1.21 (1.18-1.25)

## Data Availability

The data underlying this article will be shared on request to the corresponding author.

## Abbreviations

AB: Abacavir
AST: Aspartate aminotransferase
A_2_R: Adenosine A_2_ receptors
BMD: Bone mineral density
BS: Bone surface
BS/BV: Specific bone surface
BS/TV: Bone surface density
BV: Bone volume
BV/TV: Bone volume fraction
CAT: Catalase
Conn.D: Connectivity density
Ct.Ar: Cortical bone area
Ct.Ar/Tt.Ar: Cortical area fraction
Ct.Th: Average cortical thickness
DA: Degree of anisotropy
DFI: DNA fragmentation
DKK1: Dickkopf-related protein 1
DXA: Dual-energy X-ray absorptiometry
ET: Etravirine
GPx: Glutathione peroxidase
GSH: Reduced glutathione
GSSG: Oxidized glutathione
HDS: High DNA stainability
IHC: Immunohistochemical examination
LH: Luteinising hormone
mCT: Microcomputed tomography
MDA: Malondialdehyde
MCM-7: Minichromosome maintenance 7 protein
PINP: N-Terminal propeptide of type I procollagen
Po.tot: Total porosity
PRL: Prolactin
ROS: Reactive oxygen species
SHBG: Sex hormone binding globulin
SMI: Structure model index
SOD: Superoxide dismutase
Tb.N: Trabecular thickness number
Tb.Sp: Trabecular separation
Tb.Th: Trabecular thickness
TRACP: Tartrate-resistant acid phosphatase form 5b
TSH: Thyroid-stimulating hormone
Tt.Ar: Total cross-sectional area inside the periosteal envelope
TV: Tissue volume
1,25-(OH)_2_-D_3_: 1,25-Dihydroxyvitamin D_3_
25-OH-D: 25-Hydroxyvitamin D.
